# Effect of Square Dance Exercise on Older Women With Mild Mental Disorders

**DOI:** 10.3389/fpsyt.2021.699778

**Published:** 2021-07-29

**Authors:** Jindong Chang, Yuping Chen, Chunzhen Liu, Liming Yong, Ming Yang, Wenbing Zhu, Jibing Wang, Jiagao Yan

**Affiliations:** ^1^School of Physical Education, Southwest University, Chongqing, China; ^2^Institute of Motor Quotient, Southwest University, Chongqing, China; ^3^Qingdao Mental Health Center, Qingdao University, Qingdao, China; ^4^College of Physical Education and Health Science, Chongqing Normal University, Chongqing, China; ^5^International College of Football, Tongji University, Shanghai, China; ^6^Editorial Department, The Journal of Shandong Sports University, Shandong Sports University, Jinan, China

**Keywords:** cognition, depression, mild cognitive impairment, Chinese square dance, health

## Abstract

Many epidemiological studies have demonstrated the therapeutic benefits of exercise (EX) that can be used for adjunctive treatment in mental disorders. Despite several clinical experiments using exercise interventions, controlled studies are sparse in most disorder groups. Square dance is a popular aerobic exercise for older women in China. This study aimed to explore the effect of Chinese square dance exercise on mild mental disorders in older women. Participants included 109 older women with mild cognitive impairment from four large nursing homes. Participants were assigned either to the intervention group (*n* = 62) or the control group (*n* = 47), according to their residential nursing home. The intervention group underwent an 18-week square dance exercise, while the control group maintained their usual lifestyle. The outcomes were tested at baseline and weeks 9 and 18. The results showed that square dance exercise positively affected the results of all evaluations, especially on the participants' depressive symptoms and quality-of-life-related mental health. This study demonstrates that square dance exercise is a safe and effective approach for older women with mild cognitive impairment that benefits their long-term health.

## Introduction

Mild cognitive impairment (MCI) is a transitional state between healthy aging and dementia (1, 2). With global aging, the annual prevalence of dementia in the entire elderly population is estimated to be between 1 and 3% (3, 4), and the annual transition rate from mild cognitive impairment to dementia is estimated to be between 10 and 15%; therefore, individuals with mild cognitive impairment are at high risk of developing dementia (1). Some studies have estimated the global prevalence of mild cognitive impairment in older adults to be between 9.6 and 21.6% (5–7). The high prevalence of individuals with mild cognitive impairment and the trend toward dementia suggest that identifying effective treatments to reduce the further decline associated with cognitive abilities is necessary. Non-pharmacological interventions are considered the main approach for treating older adults with mild cognitive impairment (1). Among the various non-pharmacological treatments, physical exercise has been widely studied and promoted as a low-cost, low-risk, and easy-to-use life intervention. Several longitudinal cohort studies have shown that physical activity in midlife can prevent cognitive decline in old age (1, 8). A recent systematic review of 15 cohort studies (*n* = 33,816) showed that physical activity protects against cognitive impairment in people who are initially cognitively healthy (9). Another systematic review of prospective epidemiological studies (16 studies, 163,797 cognitively healthy participants) of dementia studies came to similar conclusions (8). Intervention studies of the effects of physical activity on cognitive performance first began in 1990, and this study showed a positive effect of aerobic walking on executive cognitive function in cognitively healthy older adults (10). Randomized controlled trials (RCTs) examining the effects of physical activity on subject cognition in healthy older adults support the claim that exercise promotes cognition in older adults (11–13). Another review study that included 11 RCTs found that aerobic exercise improved cognitive performance on tests measuring attention, delayed recall, and reaction time (14). Two subsequent studies also further confirmed that exercise had the most significant effect on executive function (11, 12). Although there is evidence that the cognitive effects of exercise are relatively consistent in cognitively healthy older adults, the impact of exercise interventions on subjects with mild cognitive impairment (MCI) is less well-understood.

The primary objective of this study was to investigate the effect of Chinese square dancing on the cognitive function of Chinese older adults with mild cognitive impairment, and the secondary purpose was to explore the impact of regular square dance exercise on health-related quality of life in older adults with MCI.

We proposed two research hypotheses. After 18 weeks of intervention, the first hypothesis was that older adult with MCI in the experimental group (i.e., square dance) would significantly improve cognitive functioning than the control group (i.e., daily lifestyle). The second hypothesis was that the experimental group would have considerably fewer depressive symptoms, better balance, and a higher quality of life than the control group.

## Methods

### Study Design and Participants

This study was a cluster-randomized controlled trial. The study sites were set in local nursing homes in Chongqing, China. Participants were enrolled in a whole-home approach with two nursing homes serving as the experimental group (EG) and two other nursing homes serving as the control group (CG). Randomization sequences were achieved by drawing lots. The sample size required for the experiment was calculated using G^*^Power software. The independent samples *t*-test was used to analyze outcomes in the change between baseline and post-experiment. With effect size (Cohen's *d* = 0.58), 80% power, and 5% Class I error, we estimated that a minimum of 48 participants per group would be required. Taking into consideration the potential dropout of participants, we recruited 10% more subjects than required; thus, each group needed to recruit a minimum of 53 participants.

We recruited participants from four pilot nursing homes recognized by the Chongqing Municipal Health and Family Planning Commission for the integration of medical and health care. These nursing homes possessed similar management patterns and bed sizes. The recruited participants met the following criteria: (1) at least 60 years old, (2) presented subjective cognitive decline in the previous year, (3) obtained a score of <26 on the Montreal Cognitive Assessment (MoCA) (plus an extra 1 point if they had achieved 12 years of schooling), and (4) attained a score of <26 on the Ability for Daily Living (ADL) assessment. Participants were excluded if they were: (1) taking medication for cognitive impairment, (2) had a neurological disorder (e.g., Parkinson's disease, stroke, multiple sclerosis), (3) had an acute or chronic condition that prevented exercise, or (4) performed regular exercise (≥30 min/day, ≥3 day/week) within the past 6 months or had sustained exercise experience for more than 5 years. All participants signed a written informed consent form before the study.

### Intervention

Chinese square dance was used for the experimental group exercise. Chinese square dance is a popular form of aerobic exercise for middle-aged and older women. The dance has a simple, easy-to-learn structure and is suitable for older individuals to use as a form of exercise. We chose dance music with simple melodies and low movement activity, and the main movement structures were hand clapping, high-fiving, chest expansion, arm extension, and leg kicking (https://www.youtube.com/watch?v=AYEasAhzHI0).

Two national social sports instructors with professional dance training taught participants Chinese square dancing over three sessions at each of the two nursing homes during the week prior to the start of the experiment. Each session lasted 1 h and was spaced 1 day apart. One of the instructors was responsible for teaching and the other for demonstrating the movements. After learning once, one instructor led the dance, and one instructor was responsible for correcting the actions until all the participants fully mastered the basic moves. Practice videos were provided after the class for everyone to familiarize themselves with the movements. During the experiment, one staff member from each nursing home was responsible for assisting with safety supervision. A sign-in system was implemented for each class during the investigation, and a small gift or daily necessities (towel, soap, toothbrush, etc.) was awarded to those participants who attended each session per week. The attendance rate for the experimental group ranged from 68.5 to 96.3%, with an average of 87.6%. Participants only missed exercise when they were unwell or did not return from an outing.

Square dance workouts were performed outdoors three times a week for 30 min each session, starting at 7 pm on Mondays, Wednesdays, and Fridays (during periods of inclement weather, sessions were held indoors). The square dance workouts at the two nursing homes were conducted in parallel. The square dance exercise was led by two researchers (national social sports instructors) each, with 5 min of warm-up activities (finger joint activities, etc.) before the training, 30 min of dancing, and 5 min of relaxation exercises (i.e., deep breathing and stretching) at the end. During the square dance exercise, it was agreed to wear a sports watch to monitor the participants' heart rate. Exercise intensity was assessed using an exercise heart rate controlled at 100–140 beats per min. The control group did not participate in the organized physical activity and led a liberal daily lifestyle.

### Outcome Measures

This study assessed participants' overall cognition, quality of life, depressive symptoms, and balance at baseline, week 9, and 18. The staff member responsible for the assessment received assessor consistency training. The assessors were blind to the group to which the participant belonged, and the trainers instructed participants not to disclose allocation information while being assessed. The primary outcome was cognitive functioning, and the rest were secondary outcomes. All scales used were in the Chinese version, and all had established evidence of reliability and validity.

The Montreal Cognitive Assessment (MoCA) was used for MCI screening in older adults (sensitivity: 90%, specificity: 83%). The MoCA contains cognitive tasks in a range of domains, including situational memory, visuospatial ability, executive function, attention, language, and orientation, to obtain an overview of a person's cognitive functioning (15). The scale has a maximum score of 30, with higher scores indicating better cognitive abilities. Similarly, the scale had a retest reliability of 0.857, and content validity, concurrent validity, and construct validity were good (*p* < 0.01) (16). The scale showed good internal consistency with a Cronbach's alpha of 0.836 (17) and was more sensitive than the Mini-Mental State Examination in measuring cognitive function in patients with mild cognitive impairment (18). In addition, the Montreal Cognitive Assessment is relatively brief compared to other complex neuropsychological combinations, making it more suitable for older adults with mild cognitive impairment.

The Short-Form 12 health survey (SF-12) was used to assess the quality of life. SF-12 is a simplified version of the SF-36 and shows high correlation with SF-36 (19). The scale includes assessing general health, physical functioning, role physical, bodily pain, vitality, social functioning, role emotional, and mental health. The first four indicators are used to assess the physical component summary (PCS), while the last four are used to assess the mental component summary (MCS). Higher scores indicate a better quality of life. For this scale, Cronbach's a was 0.775, and the criterion validity was good (*p* < 0.001) (20).

The Geriatric Depression Scale (GDS-15) was used to assess participants' depressive symptoms. The GDS-15 includes 15 items that require participants to answer “yes” or “no” for a total score of 15. Higher scores indicate more pronounced depressive symptoms, with scores >10 indicating severe depression and those >5 indicating mild depression. For the GDS-15 scale, the test–retest reliability was 0.728, and Cronbach's a was 0.793, with good discriminant validity (*p* < 0.001) (21).

The Berg Balance Scale (BBS) was used to assess participants' balance ability. The scale consists of 14 items, and each item has a score between 0 and 4 for a total score of 56, with higher scores indicating better balance ability. The inter-rater reliability of the scale was 0.992–0.998, and the retest reliability was 0.968–0.985 with good content validity (22).

### Statistical Analysis

Statistical analyses were conducted using SPSS 24.0. Tests of normality for all continuous variables were screened by Skewness and Kurtosis statistics. Descriptive statistics were used to analyze the characteristics of the participants. Means and standard deviations (SDs) were used to describe the continuous variables (e.g., age and education level of participants). Independent *t*-tests were used to compare differences between the experimental and control groups on baseline demographics and outcome variables. The paired samples *t*-test was used to compare pre- and post-experimental results within the respective experimental and control groups. To measure intervention effects over time, we used linear mixed-effects models to analyze the effects of time, group, and group-by-group time. A *p*-value of a two-sided test of <0.05 was considered to be statistically significant. Effect size estimates were calculated for all mean differences using Cohen's *d*, which relates mean score differences to pooled standard deviations (23). Data analyses were performed according to the principle of completing all experiments, and missing data were not included in the results.

## Results

### Baseline Participant Characteristics

**Figure 1:**
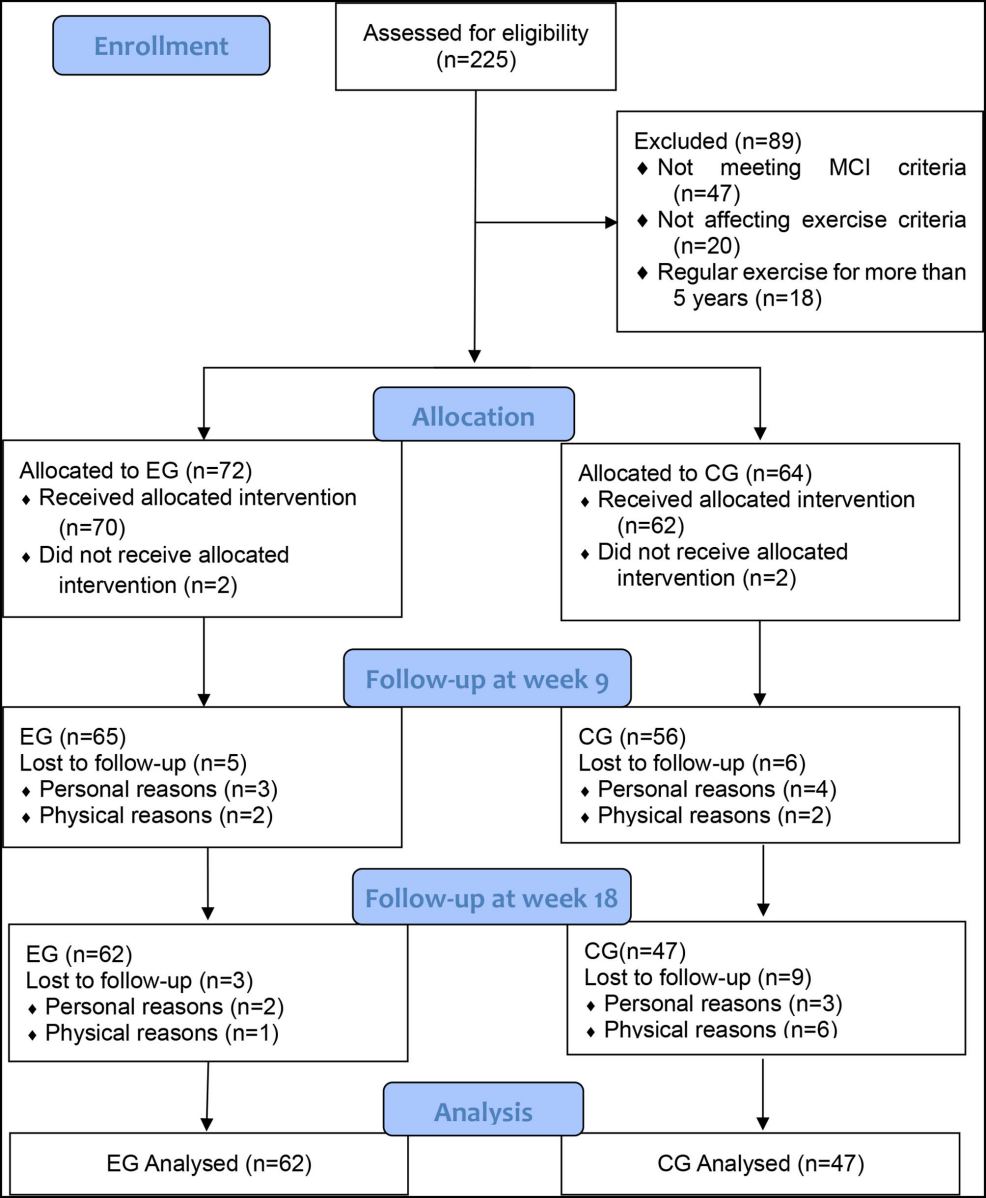
Flowchart of participant recruitment.

A flowchart of participant recruitment and withdrawal is shown in Figure 1. The participants were recruited in four nursing homes, two of which were identified as the experimental group and the other two were identified as the control group. Two hundred and twenty-five enrollments were received within 1 week. Eighty-nine individuals were excluded after initial screening, of whom 47 did not meet the MCI criteria, 20 did not meet the criteria for participation in exercise criteria, 18 had more than 5 years of regular exercise experience, and 4 did not agree to participate in the exercise protocol. The participants were grouped according to their nursing homes, with 72 in the experimental group and 64 in the control group. After 9 weeks, seven dropped out of the experimental group, two of whom did not accept the intervention program, three of whom were discharged, and two of whom were physically ill; eight dropped out of the control group, two of whom did not accept the intervention program, four of whom were released or dropped out of participation, and two of whom were physically ill. After 18 weeks, three people withdrew from the experimental group, two of whom were discharged and one who was physically ill; nine people withdrew from the control group, three of whom were discharged or dropped out of participation and six of whom were physically sick. As a result, a total of 109 participants (EG = 62, CG = 47) completed all experimental tests.

The baseline characteristics of the participants are shown in Table 1. The mean age of the participants was 76.29 ± 3.60 years, and their education level ranged from 5 to 14 years of schooling. There were no significant differences between the experimental and control groups in the baseline characteristics such as age, education level, body mass index (BMI), MoCA, BBS, PCS, MCS, and GDS scores (*p* > 0.05).

**Table 1:** Baseline characteristics of participants.

	**EG (*n* = 62)**	**CG (*n* = 47)**	***p***
Age, years	76.56 ± 3.60	75.94 ± 3.61	0.369
Education, years	8.73 ± 2.05	8.28 ± 2.06	0.261
BMI, kg/m^2^	23.63 ± 2.14	23.60 ± 2.03	0.956
MoCA	43.09 ± 6.49	42.87 ± 6.95	0.863
BBS	48.15 ± 4.97	47.11 ± 5.89	0.320
PCS	36.15 ± 5.89	35.64 ± 5.46	0.647
MCS	21.61 ± 2.11	21.49 ± 2.39	0.776
GDS	4.97 ± 1.41	4.85 ± 1.63	0.690

*Continuous variables are presented as the means ± standard deviations (SDs). BMI, Body mass index; MoCA, Montreal Cognitive Assessment; BBS, Berg Balance Scale; PCS, Physical Component Summary; MCS, Mental Component Summary; GDS, Geriatric Depression Scale*.

### Experimental Outcome Comparisons

**Figure 2:**
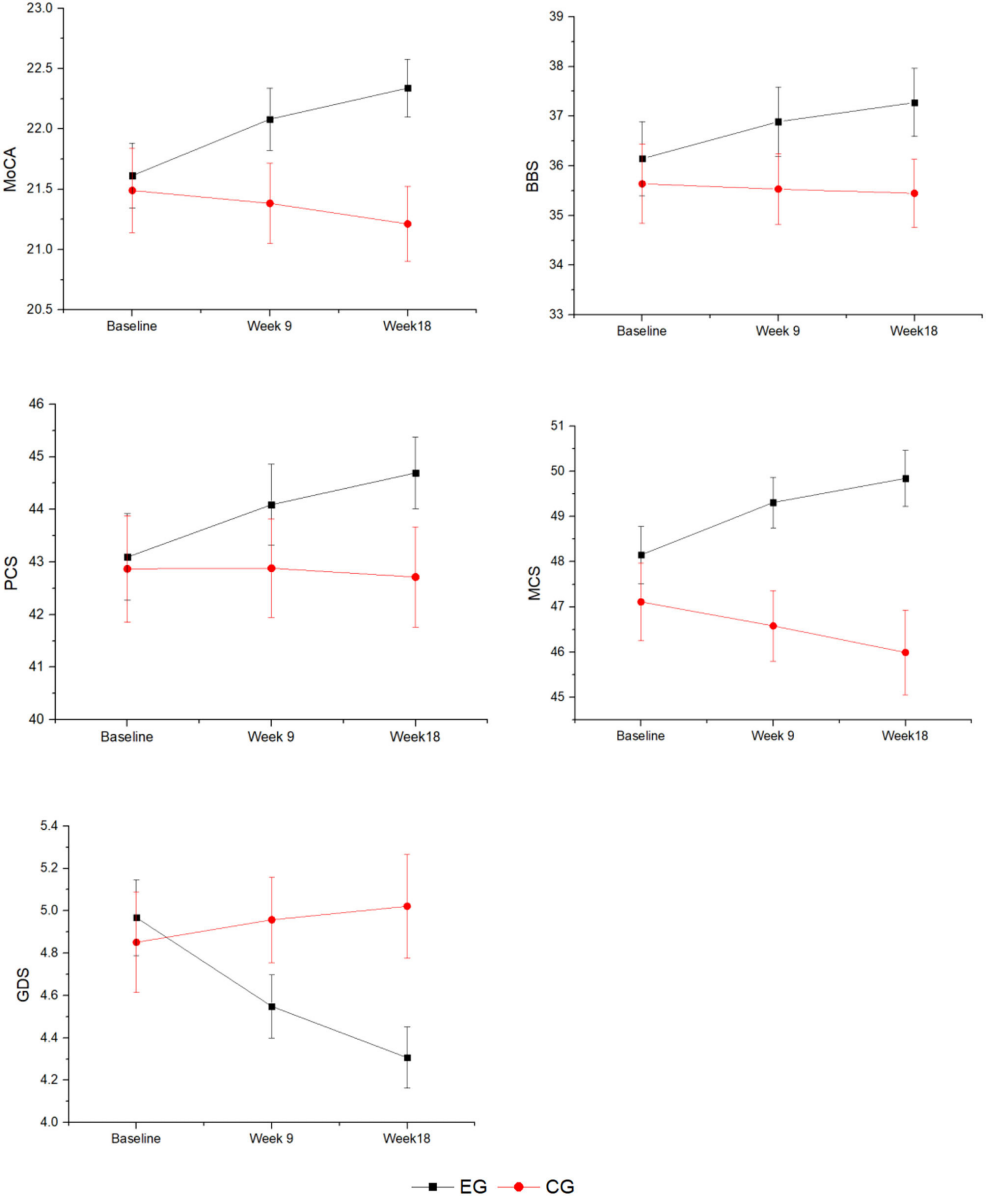
The change trend of the experimental and control groups across time. EG, experimental group; CG, control group; MoCA, Montreal Cognitive Assessment; BBS, Berg Balance Scale; PCS, Physical Component Summary; MCS, Mental Component Summary; GDS, Geriatric Depression Scale.

Table 2 presents comparisons of the results of the experimental and control groups across time. Figure 2 shows the changing trend in the experimental and control groups across time. The results of the linear mixed-effects model shows that the interaction effects of group and time were highly significant for the MoCA, BBS, and GDS (*p* < 0.001) and significant for PCS and MCS (*p* < 0.01).

**Table 2:** Comparison of the experimental and control groups across time.

**Scale**	**Time**	**EG (*n* = 62)**	**CG (*n* = 47)**	**Linear mixed-effects model (** ***p*** **)**			**Change from baseline (** ***p*** **)**		**Inter-group comparisons (*p*)**	**Effect size (*d*)**
				**Group**	**Time**	Group^*^time	**EG**	**CG**		
MoCA	Baseline	21.61 ± 2.11	21.49 ± 2.39	0.400	0.041^*^	0.776			0.776	
	Week 9	22.08 ± 2.03	21.38 ± 2.29			0.096	<0.001^***^	0.506	0.096	0.59
	Week 18	22.34 ± 1.87	21.21 ± 2.13			0.004^**^	0.001^**^	0.052	0.004^**^	0.71
BBS	Baseline	36.15 ± 5.89	35.64 ± 5.46	0.867	0.004^**^	0.647			0.647	
	Week 9	36.89 ± 5.46	35.53 ± 4.86			0.181	<0.001^***^	0.646	0.181	0.81
	Week 18	37.27 ± 5.40	35.45 ± 4.72			0.068	<0.001^***^	0.529	0.068	0.84
PCS	Baseline	43.09 ± 6.49	42.87 ± 6.95	0.620	0.011^*^	0.863			0.863	
	Week 9	44.09 ± 6.07	42.88 ± 6.40			0.318	0.001^**^	0.969	0.318	0.45
	Week 18	44.69 ± 5.35	42.71 ± 6.50			0.084	0.008^**^	0.481	0.084	0.43
MCS	Baseline	48.15 ± 4.97	47.11 ± 5.89	0.770	0.446	0.320			0.320	
	Week 9	49.31 ± 4.40	46.58 ± 5.34			0.004^**^	0.019^*^	0.364	0.004^**^	0.44
	Week 18	49.84 ± 4.86	45.99 ± 6.41			0.001^**^	0.008^**^	0.069	0.001^**^	0.63
GDS	Baseline	4.97 ± 1.41	4.85 ± 1.63	0.134	0.013^*^	0.690			0.690	
	Week 9	4.55 ± 1.17	4.96 ± 1.38			0.097	<0.001^***^	0.462	0.097	−0.56
	Week 18	4.31 ± 1.14	5.02 ± 1.67			0.009^**^	<0.001^***^	0.315	0.009^**^	−0.88

* *p < 0.05;*

** *p < 0.01;*

*** *p < 0.001*.

Inter-group comparisons at week 9 displayed a significantly higher MCS score in the experimental group (49.31 ± 4.40) than the control group (46.58 ± 5.34; *p* = 0.004). However, MoCA scores (EG: 22.08 ± 2.03; CG: 21.38 ± 2.29; *p* = 0.096), BBS scores (EG: 36.89 ± 5.46; CG: 35.53 ± 4.86; *p* = 0.181), PCS scores (EG: 44.09 ± 6.07; CG: 42.88 ± 6.40; *p* = 0.318), and GDS scores (EG: 4.97 ± 1.41; CG: 4.85 ± 1.63; *p* = 0.690) were not significantly different between the two groups. At week 18, the trend persisted with significantly higher MCS scores in the EG compared to the CG (EG: 49.84 ± 4.86; CG: 45.99 ± 6.41; *p* = 0.001), and there was a significantly lower GDS score in the EG compared to the CG (EG: 4.31 ± 1.14; CG: 5.02 ± 1.67; *p* = 0.009). Unlike week 9, at week 18, MoCA scores (EG: 22.34 ± 1.87; CG: 21.21 ± 2.13; *p* = 0.004) were also significantly higher in the EG than the CG. However, the BBS scores (EG: 37.27 ± 5.40; CG: 35.45 ± 4.72; *p* = 0.068) and PCS scores (EG: 44.69 ± 5.35; CG: 42.71 ± 6.50; *p* = 0.084) remained insignificant between the two groups.

The results of the inter-group comparison in the experimental group showed that the scores of MoCA, BBS, and PCS were significantly higher at week 9 (MoCA: *t* = 4.267, *p* < 0.001, *d* = 0.59; BBS: *t* = 5.622, *p* < 0.001, *d* = 0.81; PCS: *t* = 3.583, *p* = 0.001, *d* = 0.45) as well as at week 18 (MoCA: *t* = 3.400, *p* = 0.001, *d* = 0.71; BBS: *t* = 8.361, *p* < 0.001, *d* = 0.84; PCS: *t* = 2.752, *p* = 0.008, *d* = 0.43) compared to baseline. MCS scores were slightly higher at week 9 compared to baseline (*t* = 2.405, *p* = 0.019, *d* = 0.44) but significantly higher at week 18 (*t* = 2.721, *p* = 0.008, *d* = 0.63). GDS scores were significantly lower at week 9 (*t* = −3.681, *p* < 0.001, *d* = −0.56) and week 18 (*t* = −6.789, *p* < 0.001, *d* = −0.88) compared to baseline. In contrast, the scores of the control group at week 9 and 18 were not statistically significantly different from baseline for all outcomes (*p* > 0.05).

## Discussion

The purpose of this study was to examine the effects of 18 weeks of square dance exercise on cognitive function and health-related quality of life in Chinese older women with mild cognitive impairment and to explore its effects. This study provides evidence that Chinese square dancing has a positive impact on overall cognitive improvement, quality of life enhancement, balance enhancement, and depressive symptom regulation in older adults with MCI. To our knowledge, this is the first study in China to examine the effects of Chinese square dancing on people with MCI using southern residents as the subjects (24). We chose square dance because it is easy to learn and because it does not require specialized equipment or venues and is easily accessible, which is particularly beneficial for older participants with MCI. Good attendance and no adverse events support the feasibility of the intervention trial.

This study echoes the benefits of moderate-intensity aerobic exercise on cognitive enhancement in Chinese clinical studies (25). As for the primary outcome, the linear mixed-effects model showed a significant change in the trend of MoCA scores between the two groups. The results of the inter-group comparison showed significant improvement in the scores of the experimental group over 9 and 18 weeks, while there was no significant change in the scores of the control group. This study confirmed that over time, the intervention group exhibited enhanced cognitive function and the control group had worsening cognitive function, indicating that participants who regularly participated in physical activity had significant improvements in the same health parameters. This finding suggests that Chinese square dance as moderate-intensity aerobic exercise can serve the purpose of preventing mild cognitive impairment in older adults.

Currently, this study is consistent with most studies (26–29), although individual studies do not support the idea that aerobic exercise can improve older cognitive function (30). There are two possible reasons for this controversy. First, the small sample size (19 subjects per group) constrains the accuracy of the study results (31); second, the use of the study instrument may lead to problems with the precision of the collected data (29). The positive effects of square dancing as a form of aerobic exercise on cognitive function may be due to the presence of several factors. First, studies have shown that aerobic exercise can improve MCI in older adults (28); second, studies have shown that dance can significantly reduce depressive symptoms in older adults, and depression is an important risk factor for the development of what is known as MCI in older adults (28, 32); third, movement repetition exercises can help older adults improve their memory (33); fourth, the cordial social atmosphere and soothing musical rhythm of square dance have a positive impact on the mood of older adults, and positive mood can reduce depression, thus improving their cognitive function (34).

This study showed that square dancing had a positive effect on all results for the secondary outcomes. At week 9, the experimental group had significantly higher MCS scores than the control group. This may be because, unlike the control group, the intervention group provided more opportunities for socialization, which may have helped eliminate loneliness and improved participants' mood (35). At week 18, there was a significant difference in GDS scores between the two groups. The positive effect of square dancing on the improvement of depressive symptoms is consistent with the results of Wang et al. (23). The positive effect on physical health is supported by the fact that our study found regular, moderate-intensity square dance exercise to be promising in improving the physical condition and mental status of older adults (29).

Overall, square dancing is a promising non-pharmacological intervention strategy for older adults with MCI (36). An 18 weeks daily exercise intervention can improve overall cognition, depressive symptoms, balance, and quality of life in older MCI populations. Additionally, nursing homes and their communities can screen older adults for MCI regularly for early detection and intervention. Square dancing, an affordable form of exercise, is suitable for nursing homes, communities, and other areas where the elderly population is concentrated. Exercising in such an environment improves the mood of the elderly and is equally beneficial to improving cognition. The recommended exercise frequency is 30 min three times a week for 18 weeks; however, beyond is better. It is important to note that nursing homes or communities should provide professional assistance in supervising square dance exercises when organizing them to ensure the safety of participants.

### Limitations

This study had several limitations. First, participants were recruited from a single city and were limited by the fact that participants had to have some mild cognitive impairment, and the selected participants were not necessarily fully representative of the characteristics of people with MCI. Second, participants were from four nursing homes, and participants from each nursing home could only be coded as a whole into the experimental or control group, failing to implement a randomized controlled trial. Third, the assessment instrument used only scale measures, which had limitations. Fourth, this study only discussed MCI in older women, and the applicability to men remains to be further explored.

## Conclusion

This study investigated the effect of square dancing on exercise intervention for older women with MCI. The study supported the positive effects of square dancing in promoting cognition, depression, balance, and quality of life in MCI patients. It is recommended that Chinese square dance be conducted collectively in communities or nursing homes to promote physical and mental health and improve the quality of life of older adults.

## Data Availability Statement

The raw data supporting the conclusions of this article will be made available by the authors, without undue reservation.

## Ethics Statement

The studies involving human participants were reviewed and approved by the Scientific and Ethics Committee of Institute of Motor Quotient, Southwest University (IRB NO. SWUIMQ20180109). The patients/participants provided their written informed consent to participate in this study.

## Author Contributions

JC, YC, CL, LY, MY, and WZ: data collection. JC, LY, and JY: data analysis, conception, and design. JC, WZ, JY, and JW: research design, writing the manuscript, and revision. All authors contributed to the article and approved the submitted version.

## Conflict of Interest

The authors declare that the research was conducted in the absence of any commercial or financial relationships that could be construed as a potential conflict of interest.
